# The rates and symptoms of natural and breakthrough infection pre- and post- Covid-19 non-mRNA vaccination at various peaks amongst Iranian healthcare workers

**DOI:** 10.1186/s12985-023-02156-2

**Published:** 2023-08-18

**Authors:** Marzieh Jamalidoust, Owrang Eilami, Zahra Ashkan, Mazyar Ziyaeyan, Nasrin Aliabadi, Mohammad Habibi

**Affiliations:** 1 Department of Virology, Professor Alborzi Clinical Microbiology Research Center, Namazi Hospital, Shiraz University of Medical Sciences, Shiraz, 71937-11351 Iran; 2 Department of Family Medicine and Infectious Disease, Shiraz University of Medical Sciences, Shiraz, Iran; 3 Department of Biology, Faculty of Basic Science, Shahrekord University, Shahrekord, Iran; 4 Statistics and Information Technology Management, Shiraz University of Medical Sciences, Shiraz, Iran

**Keywords:** Breakthrough infection, Healthcare worker, Natural infection, SARS-C0V-2, Vaccination

## Abstract

**Background/Aims:**

The aim of this study was to determine the rate of natural and breakthrough infection and related symptoms of Covid-19 amongst Iranian healthcare workers (HCWs) who were vaccinated by different non-mRNA-based vaccines at peak points.

**Methods:**

In this cross-sectional study, the RT-PCR test was performed for a total of 10,581 HCWs suspicious of Covid-19 infection. For each HCW, the frequency of SARS-CoV-2 infection and the time of transmission based on vaccination administration time and schedule were examined during different waves of the pandemic. Based on these findings, the study patients were divided into three groups: natural, natural/breakthrough, and breakthrough.

**Results:**

In total, 53% of the HCWs were exposed to SARS-CoV-2 infection between 1 and 5 times within two years after the current pandemic, while 20.7% and 32.3% experienced natural and breakthrough SARS-CoV-2 infection, respectively. Only 6% of the breakthrough-infected HCWs had naturally contracted SARS-CoV-2 infection during the initial waves. The highest natural peaks of infection occurred during the interval administration of the first and second dose of the first vaccination series, while the single highest peak of breakthrough infection belonged to the Omicron wave. It occurred simultaneously with the administration of the third vaccination dose. On the other hand, the highest rate of reinfection was observed amongst people who had received the Sinopharm and Bharat vaccines full-doses.

**Conclusion:**

This study compared the clinical differences between the two peaks of Omicron and Delta. This study indicates the rates of natural and breakthrough SARS-CoV-2 infections according to vaccination schedules and different waves of the pandemic.

## Introduction

Covid-19 is caused by SARS-CoV-2 virus that went viral in the mid-December 2019. This virus and *MERS-CoV* (in 2012) as well as *SARS-CoV* (2002–2003) belong to the *Coronaviridea* family, and they have caused severe human respiratory diseases. These coronaviruses including *HCoV-229E*, *HCoV-OC43*, *HCoV-NL63*, and *HCoV-HKU1* are less pathogenic with milder symptoms in humans compared to the aforementioned ones [1, 2].

As a global health threat, Covid-19 has disproportionately affected a wide range of people working in various occupations. Healthcare workers (HCWs) who serve in this sector are the main target with potential exposure to SARS-CoV-2. Unfortunately, the HCWs in the frontlines of the battle against Covid-19 face significant risks and consequences [2, 3]. The prevention measures against Covid-19 are vital for HCWs, not only to disturb the transmission cycle, but also to maintain and sustain the risk-free work environment.

Despite restrictions imposed by the governments to reduce Covid-19 transmission and its associated morbidity and mortality in many countries, this pandemic has had devastating impacts [4]. Different solutions and approaches, such as the implementation of mass vaccination at unprecedented rate, have been proposed to end this pandemic and reduce the ever-growing concerns of Covid-19. All Covid-19 vaccines appear to be safe and effective tools to prevent severe disease, hospitalization, and death against all variants of concern, but the quality of evidence greatly varies depending on the vaccines used. Questions remain regarding the booster dose and waning immunity, duration of immunity, and heterologous vaccination. The benefits of Covid-19 vaccination outweigh the risks, despite the rare serious adverse effects [5].

Although Covid-19 vaccines are relatively effective in combating the pandemic, a small proportion of the population, especially those with comorbidities like diabetes, hypertension, cardiovascular disease, cancer, etc. who have been fully vaccinated, still develop symptomatic or asymptomatic breakthrough infection [6–8], and death may also occur in a minority. Moreover, it has been shown that the emergence of various new strains escapes the immune mechanism and can lead to vaccine failure [9–11]. A Covid-19 vaccination campaign was launched in Iran on February 9, 2021, and by the end of May 2022, almost 68% of the population received full vaccine doses.

Given the limited information on the immunogenicity of non-mRNA vaccines, we evaluated the incidence of natural and breakthrough infections in a large Iranian HCWs fully vaccinated with non-mRNA vaccines. In this cross-sectional study, we evaluated the prevalence and clinical manifestations of SARS-CoV-2 infection amongst HCWs of the hospitals affiliated with Shiraz University of Medial Sciences, Shiraz, Iran by considering the pre- and post-vaccination era based on the vaccine platforms used as well as the emergence of new strains.

## Methods

### Patients and clinical data collection

We conducted a cross-sectional study on HCWs of 36 health hospitals of the Ministry of Health, Social Security Organization, and other organizations in the city of Shiraz, Iran. The data were collected from April 2020 to February 2022.

With the onset of the outbreak, and given the seriousness of Covid-19 threats, the Iranian Ministry of Health took the necessary measures to manage the disease. Hence, the clinical and lab data of the Covid-19 patients and the “corona lab” data bases were stablished in the Iranian universities of medical sciences. Shiraz University of Medical Sciences (SUMS) is one of the largest in Iran with more than 30,000 HCWs including employees, students, and volunteers. In the current study, 10,581 HCWs showed at least one respiratory symptom (fever, cough, rhinorrhea, etc.) and underwent PCR test as suspected Covid-19 infected patients during that period.

Eligibility criteria for inclusion in this study were being 18 years or older, being affiliated with SUMS, and being vaccinated with any of the AstraZeneca, Sinopharm, Sputnik, Baharat, Spiko Gene, CovIran Barekat, and Soberna vaccines or not receiving any Covid-19 vaccination during that period.

The type of infection, as the main outcome variable, was assessed in the current study. Infected HCWs were divided into three groups based on individual-level data and the timing of infection and vaccination schedule. ‘Natural’ type individuals were patients infected with Covid-19 prior to receiving the vaccine. ‘Breakthrough’ infected people were those infected with Covid-19 after vaccination, and ‘natural/breakthrough’ infected ones were those infected twice or more with the virus before and after vaccination.

The HCWs clinical and laboratory data were collected and then processed through the http://coronalab.sums.ac.ir/ until February 25, 2022. All the corresponding demographic data including sex, age, illness onset date, diagnosis date, residency location, clinical symptoms, and hospital reports were recorded in the aforesaid website.

### SARS-CoV-2 detection by real-time reverse transcription polymerase chain reaction (rRT-PCR)

Naso- and oro-pharyngeal swabs were collected from each symptomatic HCWs using Dacron swab and sterile VTM; from them, 200 μL of the samples were extracted using Sina Pure™ Viral (EX6061, Tehran, Iran) and BehPrep (AAAA15044000, Tehran, Iran) viral nucleic acid extraction kits. Reverse transcriptase– Taq-Man real time polymerase chain reaction (RT-PCR) was performed for each patient using different commercial kits (Pishtaz Teb PT. COVID.100 and Topaz TGK2005-100, Tehran, Iran). The detection limit of this test is 200 copies per milliliter. To detect SARS-CoV-2 RNA, we used two different RT-PCR systems, namely rotor-gene Q (Berlin-Germany) and ABI one Step plus (USA). This study was approved by ethics committee of SUMS, Shiraz, Iran (IR.SUMS.REC.1399.1319).

### Statistical analysis

Data were analyzed, using IBM SPSS version 26. The results were expressed in frequency and proportion for categorical variables as well as mean and standard deviation for continuous variables. The significance of the difference between proportions was assessed using the chi-square test. A p-value < 0.05 was considered as statistically significant.

## Results

The age range of the participants was between 19 and 75 years with a mean of 34.33 years (SD ± 7.638) (95% CI: 34.18–34.49). A total of 76.1% of the infected HCWs were under 40 years. The male/female ratio was 0.52. Out of more than 30,000 health workers, 10,581(35.27%) (95% CI: 34.7–35.8) were symptomatic and experienced at least one respiratory symptom (fever, cough, rhinorrhea, etc.) and underwent SARS-CoV-2 RT-PCR testing. Most infected HCWs were women (65.7%)(95% CI: 64.8–66.6). During the study, 4562, 952, 86, 11, and 1 infected HCWs became positive between 1 and 5 times, respectively.

### Natural, breakthrough and natural/breakthrough infection rate among HCWs

A total of 4969 (47%) (95% CI: 46-47.9) symptomatic HCWs did not experience SARS-CoV-2 infection during the study period, while among the remaining 53% infected HCWs, 2195 (20.7%) (95% CI: 20-21.5) experienced natural infection 1 to 3 times in the pre-vaccination era and never experienced SARS-CoV-2 infection during post-vaccination period;632(6%) (95% CI: 5.5–6.4) HCWs who were naturally infected got infected again in the ensuing waves even after receiving the full dose of the vaccines shot. This group is referred to as Natural/Breakthrough infected individuals in this study. The last group consisted of 2785 (26.3%) (95% CI: 25.5–27.2) breakthrough infected HCWs who were not infected with Covid-19 in the pre-vaccination era, but became exposed to the SARS-CoV-2 infection during the post-vaccination period during the latest waves (Table 1). The mean age in the infected groups was not statistically significant.

**Table 1:** Pattern and frequency of SARS-CoV-2 infection in natural, natural/ breakthrough, and breakthrough infected healthcare workers, Shiraz, Iran. Minus and plus signs indicate infection pre- and post-receiving of the full dose of vaccination, respectively (N = 5612)

Number of SARS-CoV-2 positive test	Natural infected HCWs - [N = 2195(%)]	Natural/ Breakthrough infected HCWs -/+ [N = 632(%)]	Breakthrough infected HCWs + [N = 2785(%)]
1	-1 [N = 2056(93.7%)]	……	+ 1 [N = 2498(89.7%)]
2	-2 [N = 129(5.9%)]	-1/+1[ N = 552 (87.3%)]	+ 2 [N = 279 (10%)]
3	-3[ N = 10(0.4%)]	-2/+1 [N = 38 (6%)] -1/+2 [N = 31 (4.9%)]	+ 3 [N = 7(0.27%)]
4	……	-2/+2[ N = 4 (0.6%)] -3/+1[ N = 6(1%)]	+ 4 [N = 1(0.03%)]
5	……	-2/+3 [N = 1(0.2%)]	……

Abbreviation: HCW = healthcare worker

Data are presented as case number (percentages)

HCWs who were naturally infected were denoted with a negative sign, and the number next to it indicated the number of infections; similarly, those who had a natural/breakthrough SARS-CoV-2 infection were denoted with a -/+ sign, and the number next to it indicated the number of infections SARS-CoV-2 before/after vaccination; and finally, those who had the breakthrough form are denoted with a positive sign, which denotes the number of times the person contracted the infection after receiving the vaccine

### Vaccination schedule and timing of SARS-CoV-2 infection in HCWs

**Fig. 1:**
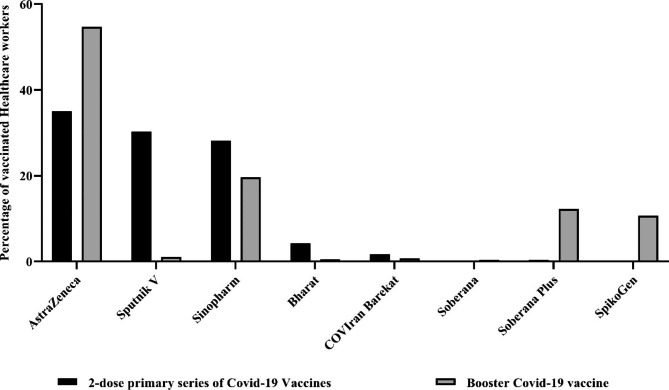
Primary immunization program (two doses) and heterologous booster vaccination and the proportion of each vaccine among Iranian healthcare workers, who experienced SARS-CoV-2 infection (N = 5612)

It is worth mentioning that in the first vaccination series of 5612 infected HCWs with different types of SARS-CoV-2, 99.1% of doses one and two were homologous, while after dose three, only 42.6% were heterologous. AstraZeneca was the most widely administered vaccine for primary and booster scheduled vaccination (Fig. 1).

**Fig. 2:**
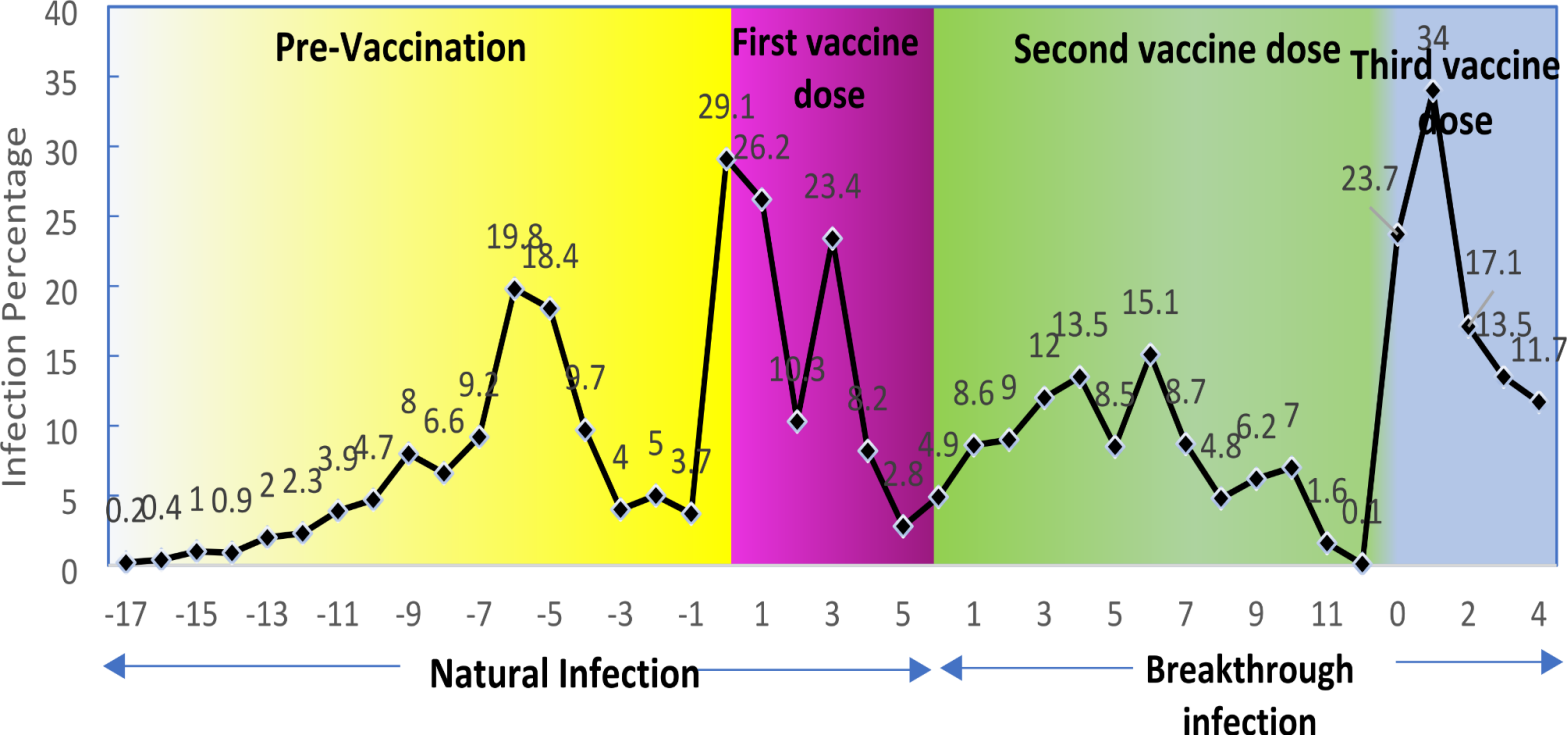
Individual-level time patterns during 17 months before the onset of the vaccination campaign (yellow box) up to five months after the first vaccination administration (purple box) determined as natural SARS-CoV-2 infections about 14 days up to 11 months after dose 2 vaccination administration (green box) and up to 4 months after administration of booster dose (blue box), indicating SARS-CoV-2 breakthrough infection among Iranian healthcare workers between April 2020 and Feb 2022

In this cross-sectional study, the natural infection rates occurred within 14 days after receiving full vaccination doses. The respective symptoms and the rate of breakthrough infections were assessed 14 months after receiving full vaccine doses amongst the HCWs. As shown in Fig. 2, the two highest peaks of the natural infection were in line with receiving the first dose and the time interval between the two doses (29.1% and 23.4%, respectively). This mainly occurred during Beta and Delta waves, followed by 6 months before the first dose of the vaccine during the Alpha wave (19.8%). The main peak of the breakthrough infection coincided with the onset of the Omicron wave in December 2021, which increased significantly to over 34%. This was much higher than the prevalence of other infection peaks in the previous waves.

### Natural and breakthrough infection rate of HCWs in different pandemic waves

Overall, 50% (N = 1709) of the breakthrough infections in HCWs occurred in the Omicron wave, while 26.7% (N = 911) occurred in the Delta and 23.3% (N = 797) in other waves. Furthermore, out of the 2195 naturally infected HCWs, 20% (N = 439), 63.4% (N = 1392), 12.2% (N = 268), and 4.4% (N = 96) had experienced infection during Wuhan, Alpha, Beta, and Delta waves, respectively. No naturally infected HCW was recorded in the Omicron wave.

### The incidence of breakthrough infections according to the vaccination schedule and the pandemic wave

The breakthrough infection rate varied after using different vaccines, while the highest breakthrough rate for all vaccination schedules occurred with the Omicron wave and, in this case, “Cov-Iran Barekat” vaccine had the highest rate (22.6%). Overall, the highest rate of breakthrough infections was observed after the use of Bharat and Sinopharm vaccines at a rate of approximately 35% although no statistically significant differences were demonstrated between the different vaccination programs (Table 2).

**Table 2:** Prevalence of natural/breakthrough and breakthrough infections after administration of different vaccination platforms among Iranian health workers who had received full dose vaccination during different SARS-CoV-2 waves (N = 10,533)

Full dose vaccine schedule	Natural/ Breakthrough infected HCWs N = 632		Breakthrough infected HCWs N = 2785			Total Number 3417 (32.4%)	P value
	Another waves/Omicron wave N = 616 (97.5%)	Delta/ Omicron Wave N = 16 (2.5%)	Other waves N = 181 (6.5%)	Delta Wave N = 895 (32.1%)	Omicron Wave N = 1709 (61.4%)		
AstraZeneca N = 3420 (32.46%)	237 (6.9%)	4 (0.12%)	40 (1.2%)	303 (8.8%)	574 (16.8%)	1158 (33.85%)	**< 0.001**
Sinopharm N = 2803 (26.6%)	137 (4.9%)	9 (0.32%)	74 (2.6%)	216 (7.7%)	546 (19.5%)	982 (35.03%)	**< 0.001**
Sputnik N = 3694 (35.1%)	212 (5.7%)	-	48 (1.3%)	319 (8.6%)	483 (13.1%)	1062 (28.75%)	**< 0.001**
Baharat N = 449 (4.27%)	18 (4%)	-	19 (4.2%)	54 (12%)	70 (15.6%)	161 (35.85%)	**< 0.001**
Spiko Gene N = 4 (0.03%)	-	-	-	-	-	-	**-**
CovIran Barekat N = 159 (1.5%)	12 (7.5%)	3 (1.9%)	-	3 (1.9%)	36 (22.6%)	54 (33.96%)	**< 0.001**
Soberna N = 4 (0.04%)	-	-	-	-	-		**-**
Total 10,533^1^(100%)	616 (5.8%)	16 (0.15%)	181 (1.7%)	895 (8.5%)	1709 (16.2%)	3417 (32.44%)	

^1^ Dose 1 and 2 were different in 48/10,581 suspected HCW

### Clinical presentation in SARS-CoV-2 breakthrough vs. naturally infected HCWs

As shown in Table 3, sore throat in the breakthrough-infected HCWs was significantly more frequent than that of natural and non- SARS-CoV-2 infected patients(P value ≤ 001). Additionally, fever, shortness of breath, and runny nose were the other symptoms significantly more common in infected HCWs (p value ≤ 0.05).

As revealed in Table 4, fever, cough, sore throat, runny nose, headache, dizziness, fatigue, and general weakness of the body were less common during Delta wave as compared to Omicron waves.

**Table 3:** Comparison of symptoms in natural and breakthrough SARS-CoV-2 positive and negative healthcare workers, Shiraz, Iran (N = 10,581), (* p < 0.05, **** p < 0.0001)

Symptoms	Non infected HCWs N = 4969	Natural Infected HCWs N = 2195	Breakthrough infected HCWs N = 2785	Natural/ Breakthrough infected HCWs N = 632	P Value
Fever	888 (17.9%)	379 (17.3%)	559 (20.1%)	112 (17.7%)	**0.043**
Cough	1266 (25.5%)	554 (25.2%)	752 (27%)	170 (26.9%)	0.384
Sore throat	1395 (28.1%)	540 (24.6%)	931 (33.4%)	165 (26.1%)	**0.000**
Runny nose	655 (13.2%)	270 (12.3%)	416 (14.9%)	77 (12.2%)	**0.030**
Shortness of breath	502 (10.1%)	225 (10.3%)	238 (8.5%)	73 (11.6%)	**0.043**
ARDS	3 (0.1%)	2 (0.1%)	3 (0.1%)	1 (0.2%)	0.818
Severe pneumonia	2 (0.04%)	0	1 (0.03%)	0	0.776
Mild pneumonia	5 (0.1%)	0	3 (0.1%)	1 (0.2%)	0.462
Nausea	238 (4.8%)	89 (4.1%)	133 (4.8%)	29 (4.6%)	0.558
Diarrhea	246 (5%)	105 (4.8%)	143 (5.1%)	46 (7.3%)	0.076
Abdominal pain	106 (2.1%)	51 (2.3%)	74 (2.7%)	20 (3.2%)	0.268
Chest pain	114 (2.3%)	69 (3.1%)	69 (2.5%)	19 (3%)	0.173
Headache	1003 (20.2%)	464 (21.1%)	558 (20%)	132 (20.9%)	0.753
Dizziness	183 (3.7%)	76 (3.5%)	95 (3.4%)	24 (3.8%)	0.906
Myalgia	431 (8.7%)	187 (8.5%)	241 (8.7%)	57 (9%)	0.984
Fatigue	1492 (30%)	651 (29.7%)	899 (32.3%)	197 (31.2%)	0.140
General weakness of the body	813 (16.4%)	370 (16.9%)	457 (16.4%)	107 (16.9%)	0.946

**Table 4:** Association between SARS-CoV-2 variants of concern (Delta or Omicron) type and the occurrence of different respiratory symptoms in Iranian healthcare workers that were tested positive for SARS-CoV-2 infection (p < 0.05)

Symptoms	Delta Wave (N = 1014)	Omicron Wave (N = 1709)	P Value
Fever	179 (17.7%)	428 (25%)	**0.000**
Cough	248 (24.5%)	562 (32.9%)	**0.000**
Sore throat	263 (25.9%)	808 (47.3%)	**0.000**
Runny nose	130 (12.8%)	633 (37%)	**0.000**
Shortness of breath	95 (9.4%)	141 (8.3%)	0.316
ARDS	1 (0.1%)	3 (0.2%)	0.523
Severe pneumonia	0	1 (0.1%)	0.628
Mild pneumonia	1 (0.1%)	2 (0.1%)	0.687
Nausea	51 (5%)	99 (5.8%)	0.399
Diarrhea	66 (6.5%)	91 (5.3%)	0.200
Abdominal pain	36 (3.6%)	46 (2.7%)	0.205
Chest pain	29 (2.9%)	47 (2.8%)	0.866
Headache	204 (20.1%)	768 (44.9%)	**0.000**
Dizziness	31 (3.1%)	99 (5.8%)	**0.001**
Myalgia	101 (10%)	192 (11.2%)	0.300
Fatigue	303 (29.9%)	700 (41%)	**0.000**
General weakness of the body	156 (15.4%)	333 (19.5%)	**0.007**

## Discussion

Based on PCR testing, the present study revealed that 53% of symptomatic HCWs had 1 to 5 times SARS-CoV-2 infections within 2 years after the pandemic, while 20.7%, 26.3%, and 6% experienced natural, breakthrough, and natural/breakthrough SARS-CoV-2 infection, respectively. To the best of our knowledge, this study is the first to assess the SARS-CoV-2 infection rate of HCWs infected with the virus before and after vaccination.

Although the prevalence of natural SARS-CoV-2 infection in HCWs, based primarily on PCR testing, is 11%, various studies have shown that the United States (55%), Mexico (30.35%), China, Denmark, and Italy (49.3%) have the highest levels of SARS-CoV-2 infection. In contrast, Kuwait (20.5%), Egypt (20%), Canada (7%), and Austria reported the lowest number of HCWs with SARS-CoV-2 infections [12]. As reported in the present study, this figure was 20.7% for the natural infection in Iran. Certainly, further differences stated might be due to the differences in sample size, methodology, and geographic diversity.

In this study, we also investigated Covid-19 breakthrough infections among 10,581 fully vaccinated HCWs during four months after the administration of the third vaccine dose. Vaccination-induced protection against the disease is expected to decrease over time [13], and different levels of immunity are expected to impact susceptibility to breakthrough infections. In this study, a total of 32.3% of the symptomatic HCWs experienced breakthrough infection, out of whom 6% had been exposed to the natural infection prior to receiving their vaccination. The major peak of breakthrough infections coincided with the onset of the Omicron wave, which was higher than the prevalence of others in previous waves, likely due to insufficient immunity and poor protections as the result of high confidence in receiving the vaccine dose. Overall, 50% of the breakthrough infections in HCWs occurred in the Omicron wave, while in the study carried out by Sormani et al., 75% of breakthrough infections happened in the Omicron wave; this is in line with the findings of our study [14].

After using different vaccines, many authors reported different rates of breakthrough infection. Various figures were reported on the prevalence of breakthrough infections among those who received the full dose of Moderna and Pfizer’s vaccines. Jara et al.’s study showed that, compared to no vaccination, adjusted vaccine efficacy with homologous booster was 78.8% against laboratory-confirmed SARS-CoV-2 infection, while heterologous BNT162b2 booster was 96.5% and AZD1222 booster was 93.2% [15]. In a study, Patalon and her colleagues estimated 70–84% reduction in the likelihood of testing positive for SARS-CoV-2 infection among people who received a BNT162b2 booster dose [16]. Another study found that the infection rate in the booster group was 11.3 times lower in the breakthrough infected subjects [17].

Studies in India after receiving the AZD1222 (ChAdOx1S) or BBV152 vaccine reported a breakthrough infection rate of just over 13% [18, 19]. The reported that the data in these studies were related to the prevalence of the Delta variant, but not for Omicron, while a preliminary study conducted in India, which coincided with the prevalence of the Beta variant, reported the breakthrough infection rate at only 1.6% [20]. Several studies estimated that the breakthrough infection rate in China was 15-20% [21–23].

Determining the frequency, severity, and cause of breakthrough infections can influence the health system response capabilities. A timely follow-up is vital when breakthroughs are relatively rare or mild, and the infection rate is not expected to increase significantly. The importance of breakthrough infections in HCWs with or without previous exposure to natural infection is exacerbated when significant changes occur in severity and frequency, especially during the emergence of new virus strains [24, 25]. Several studies have highlighted the reduction in hospitalizations, mortality, and ICU admission rates in addition to a reduction in the disease severity in people with breakthrough infections [16, 17, 26, 27]. In this study, the data revealed that the prevalence of breakthrough infection among HCWs during the Covid-19 Omicron wave was very high, but the severity of the disease decreased significantly; this is consistent with the findings of a previous study [3]. Furthermore, nearly one in three HCWs experienced a breakthrough infection after receiving both scheduled doses of the Covid-19 vaccine. These findings suggest that in the real-world settings a significant proportion of the vaccinated individuals with a high risk of exposure and those with comorbidities remain vulnerable to Covid-19 infection albeit with reduced disease severity in most cases.

The symptoms that characterize Omicron infection moderately differ from those of the Delta SARS-CoV-2 variant. Headache, runny nose, and sore throat were the symptoms with the highest percentage of difference in the incidence rate between Omicron and Delta waves that have affected symptomatic patients with 24.8%, 24.2%, and 21.4%, respectively. Our study is in line with those of *Kim et al.* [28]. and *Davies et al.*.’s [29].studies; however, in *Menni et al.*’s. study, runny nose was reportedly more prevalent in Delta waves compared to Omicron waves [30].

One of the main limitations of this study was lack of access to information on mortality, hospitalizations, and ICU admission of the HCWs. Another limitation of this study was lack of access to the clinical presentation and lab data of patients with natural and/or breakthrough infections, especially serum iron, transferrin, and ferritin, which could determine the role of these factors in severity of different forms of the disease [31].

The findings of the present study indicate the rates of natural and breakthrough SARS-CoV-2 infections among Iranian HCWs within two-year post-pandemic era in Iran. Moreover, the clinical symptoms amongst people affected with different forms of infection (natural vs. breakthrough) as well as different waves were compared. Vaccination schedules in breakthrough-infected healthcare workers were also evaluated. Multipronged prevention strategies are needed to reduce Covid-19-related morbidity and mortality.

## Data Availability

The data that support the findings of this study are available on request from the corresponding author.
